# QTL mapping for early root and shoot vigor of upland rice (*Oryza sativa* L.) under P deficient field conditions in Japan and Madagascar

**DOI:** 10.3389/fpls.2022.1017419

**Published:** 2022-10-24

**Authors:** Harisoa Nicole Ranaivo, Dinh Thi Lam, Yoshiaki Ueda, Juan Pariasca Tanaka, Hideki Takanashi, Landiarimisa Ramanankaja, Tantely Razafimbelo, Matthias Wissuwa

**Affiliations:** ^1^ Rice Research Department, National Center for Applied Research on Rural Development (FOFIFA), Antananarivo, Madagascar; ^2^ Crop, Livestock and Environment Division, Japan International Research Center for Agricultural Sciences (JIRCAS), Tsukuba, Japan; ^3^ Institute of Agricultural Science for Southern Vietnam (IAS), Ho Chi Minh City, Vietnam; ^4^ Graduate School of Agricultural and Life Sciences, The University of Tokyo, Tokyo, Japan; ^5^ Laboratory of Radioisotopes, University of Antananarivo, Antananarivo, Madagascar

**Keywords:** phosphorus deficiency, crop establishment, crown root number, NERICA, seedling vigor

## Abstract

Upland rice production is limited by the low phosphorus (P) availability of many highly weathered tropical soils and P deficiency is likely to become increasingly limiting in future drier climates because P mobility decreases sharply with soil moisture. Good seedling root development will be crucial to cope with the combined effects of low P and water availability. Upland rice genebank accession DJ123 was used as a donor for P efficiency and root vigor traits in a cross with inefficient local variety Nerica4 and a set of backcross lines were used to characterize the seedling stage response of upland rice to low P availability and to identify associated QTL in field trials in Japan and Madagascar. Ten QTL were detected for crown root number, root, shoot and total dry weight per plant in a highly P deficient field in Japan using the BC_1_F_3_ generation. Of these, *qPef9* on chromosome 9 affected multiple traits, increasing root number, root weight and total biomass, whereas a neighboring QTL on chromosome 9 (*qPef9-*2) increased shoot biomass. Field trials with derived BC_1_F_5_ lines in a low-P field in Madagascar confirmed a highly influential region on chromosome 9. However, *qPef9-*2 appeared more influential than *qPef9*, as the shoot and root biomass contrast between lines carrying DJ123 or Nerica4 alleles at *qPef9-*2 was +23.8% and +13.5% compared to +19.2% and +14.4% at *qPef9*. This advantage increased further during the growing season, leading to 46% higher shoot biomass at the late vegetative stage. Results suggest an introgression between 8.0 and 12.9 Mb on chromosome 9 from P efficient donor DJ123 can improve plant performance under P-limited conditions. The QTL identified here have practical relevance because they were confirmed in the target genetic background of the local variety Nerica4 and can therefore be applied directly to improve its performance.

## Introduction

The growing global population necessitates continuous increases in crop production and this need is especially urgent for rice in Sub-Saharan Africa (SSA) where a second trend, the shifting consumer preferences away from traditional crops towards rice consumption, has caused local rice production to increasingly fall behind local rice demand (Nigatu et al., 2017). The total rice cultivation surface harvested in SSA was around 11.2 million hectares in 2016 (Nigatu et al., 2017) and that included an expansion of the upland rice area by more than 1 million ha in Africa (Saito et al., 2018). Upland rice in SSA is predominantly grown by subsistence farmers that either lack the financial resources to purchase off-farm inputs such as fertilizers or may decide not to apply fertilizers as a risk-avoidance strategy given that upland rice productivity fluctuates strongly with year-to-year variation in rainfall patterns (Saito et al., 2018). As a result upland rice yields are typically far below those achieved in irrigated lowland rice (van Oort et al., 2015; Saito et al., 2018) and the vulnerability of upland rice production is expected to further increase with global climate change as rainfall patterns are expected to become more irregular (Wassmann et al., 2009; Heredia et al., 2021).

Upland rice is typically grown on the highly weathered soils of the humid tropics that strongly sorb P due to the abundance of iron and aluminum oxides in their clay fractions, rendering P largely unavailable to plant roots (Alewell et al., 2020). P deficiency is therefore considered to be the most important yield-limiting factor in upland rice production of SSA together with drought (Saito et al., 2018). P deficiency is likely to be increasingly limiting in future drier climates because P mobility decreases sharply with the decline of soil moisture (Marin et al., 2021). This would particularly affect seedling establishment and early crop development when roots are developing and soil exploration by roots is not sufficiently large to acquire the immobile P. To overcome P limitations during this early phase, one low-cost strategy would be to apply a micro-dose of P fertilizer together with seeds into the planting hole (Rakotoson et al., 2020), while exploring genotypic variation for early seedling vigor during P deficiency would represent a second option.

Genotypic differences in P uptake by rice plants do exist and are mainly caused by genotypic differences in root growth rates and to a lesser extent by differences in the quantity of P acquired per root size, or root efficiency (Mori et al., 2016; Wissuwa et al., 2020). It was furthermore shown that differences in P acquisition ability exist during the early seedling stages that are driven by rapid seedling root development (Pariasca-Tanaka et al., 2015). It would thus be beneficial to explore such traits leading to better crop establishment in rice breeding and this study attempts to do so for upland rice in Madagascar.

Nerica4 is a popular upland rice variety in Africa including Madagascar due to its good grain quality, good response to fertilizer, tolerance to drought stress and resistance to the parasitic weed striga (WARDA, 2001). However, Nerica4 does not perform well on infertile soils in the absence of fertilizers (Atakora et al., 2015; Vandamme et al., 2016). This poor performance may be caused in part by its low capacity to acquire P from P-sorbing soils (Koide et al., 2013; Wissuwa et al., 2020). In contrast, the genebank accession DJ123 of Bangladeshi origin and belonging to the aus sub-species of rice was identified as being well-adapted to low-P African soils, especially if P deficiency coincided with water limitations (Vandamme et al., 2016). Subsequently it was shown that DJ123 has much faster seedling root development than Nerica4 and that it combines early root growth with superior root efficiency and internal P utilization efficiency (Wissuwa et al., 2020), making it a potential donor for these desired traits.

Quantitative trait loci (QTL) for performance under P deficient conditions had been identified earlier in rice (Ming et al., 2000; Wissuwa et al., 2002; Mu et al., 2003; Shimizu et al., 2004) and of these, only the large-effect QTL *Pup1* had been narrowed down to a single gene (OsPSTOL1; Gamuyao et al., 2012) and was subsequently utilized in marker-assisted selection to develop rice varieties with improved performance under P deficiency (Swamy et al., 2020). Many modern varieties developed for favorable lowland conditions completely lack the entire Pup1 locus including OsPSTOL1, but both Nerica4 and DJ123 carry OsPSTOL1 alleles (Pariasca-Tanaka et al., 2014, and unpublished data). To identify novel loci that further improve seedling vigor under P deficiency beyond the level conferred by Pup1, it may thus be desirable that both parents of the mapping population were carriers of OsPSTOL1.

The present study utilized a QTL mapping population derived from P efficient donor DJ123 and inefficient recurrent parent Nerica4 with the aim to identify novel loci controlling early vigor traits under P deficient conditions. We used a back-cross population to minimize potentially negative effects of exotic donor DJ123. Specifically, our objectives were to perform QTL mapping in a BC1F3 population on a highly P-sorbing soil in Japan in the absence of water limitations; and to confirm identified QTL in field trials in Madagascar and Japan using selected homozygous lines in the BC1F5 generation.

## Materials and methods

### Plant material

An initial breeding population targeting P deficient upland environments in SSA had been developed from a cross of P efficient genebank accession DJ123 with P inefficient upland variety Nerica4 by the Africa Rice Center and shared with FOFIFA for further advancement and evaluation in Madagascar. DJ123 belongs to the aus subpopulation of rice whereas Nerica4 had been developed from an interspecific cross between an *Oryza glaberrima* donor and recurrent parent WAB56-104 belonging to the tropical japonica subpopulation of *Oryza sativa*. From the initial DJ123 x Nerica4 population, line NDJ188 was found to have good field performance in Madagascar and was back-crossed to Nerica4 to develop a BC1 QTL mapping population of 201 lines. These were genotyped in the BC1F2 and phenotyped in the derived BC1F3 generation. The confirmation of detected QTL was done in the BC1F5, which was developed by advancing one single selected BC1F3 plant to the F4, where another single selected plant was used as the founder of the BC1F5.

### Phenotyping under hydroponic and rhizobox conditions

Parents (DJ123, Nerica4 and NDJ188) were characterized through nutrient solution and rhizobox experiments. Plants were grown with Yoshida nutrient solution (Yoshida et al., 1972) in which the standard P concentration of 320 μM P was reduced to 2 μM P to evaluate plant growth under P deficiency. The rhizobox experiment used the same low-P soil described for field experiment 1 below. The dimensions of the plexiglas rhizoboxes were 30 x 30 x 3 cm (height x width x depth for outer dimensions). Plants in both experiments were sampled at 35 days after sowing (DAS). The number of crown roots (RN) was counted and root dry weight (RW) and shoot dry weight (SW) were determined after oven-drying samples at 65°C for 3 days. Total dry weight per plant (TW) was calculated from the sum of RW and SW.

### Phenotyping under field conditions

Three field experiments were conducted in Japan and Madagascar over the 2020-2022 period.

#### Experiment 1

The 201 BC1F3 lines were evaluated at the experimental field station of JIRCAS in Tsukuba, Japan (36°05’N, 140°08’E; altitude 50 m above sea level) in 2020 in an upland field plot that had not been fertilized with P for more than 15 years. The soil at the site is a volcanic ash soil (Humic Haplic Andosol) with a pH of 5.8 and very high P fixing capacity, leading to a very low level of plant available P (5 mg P kg^−1^ soil, Bray-II). Before sowing, basal NPK fertilizer was applied at the recommended rate of 50-0-50 kg/ha. Parents and 201 BC1F3 lines of the mapping population were sown in a randomized complete block design with four replications on 16th June, 2020. Each entry was sown in double-row microplots with spacing of 15 cm between rows and 10 cm within rows. The length of each row was 2 m. The field was occasionally supplied with water to prevent drought stress and weeds were manually removed. Three plants per replicate plot were sampled 40 DAS (n=12). The evaluated traits were RN, RW, SW, and TW. Plants were dug out to a depth of 20 cm using a spade and soil was washed off with water. The number of crown root per plant was counted, then shoot and root were separated, dried in the oven for 3 days at 70°C and weighed. Total dry weight was obtained by adding RW and SW.

#### Experiment 2

BC1F5 lines were used in 2021 to confirm the QTLs detected in 2020 in the BC1F3 population. Experiment 2 was sown at JIRCAS in Tsukuba on June 2nd, 2021 in a neighboring field with slightly higher P availability (7 mg P kg^−1^ soil, Bray-II) but also without fertilizer-P application (N and K were applied as in 2020). BC1F5 lines were sown in a randomized complete block design with two replications in 2-row microplots as described above and 3 plants were sampled per plot (n=6). Sampling was done 40 DAS using same procedures as above.

#### Experiment 3

BC1F5 lines were furthermore evaluated in Ankazo, Madagascar (19° 39’S 46° 30’E; altitude 1016 m above sea level), to confirm the efficacy of detected QTL in a very different environment. Fields in Ankazo were unfertilized following typical farmers practice in the region. The soil at the site is classified as Oxisol/Ferrasol, with a pH of 5.1 and a low level of plant available P of 6.63 mg P kg^−1^ soil (Olsen). Lines were sown in double-rows, with a spacing of 10 cm between rows and 10 cm within rows, on February 8th, 2022. The experiment was not replicated but 5 plants per plot were sampled 40 and 70 DAS from the same lines as in Experiment 2, considering the AA and BB alleles of the detected QTL on chromosome 9.

### Molecular marker detection through restriction site associated DNA sequencing (RAD-seq)

DNA was extracted from leaves by the standard phenol-chloroform method, and genotype data were obtained using restriction site-associated DNA sequencing (RAD-seq) (Baird et al., 2008). The detailed methods and information for library construction for RAD-seq were described in Kobayashi et al. (2017). The resultant library was sequenced with the Hiseq-X instrument (Illumina). The quality of reads was analyzed by FastQC software (ver 0.11.9) (https://www.bioinformatics.babraham.ac.uk/projects/fastqc/). The raw sequence reads generated were trimmed for remaining adapters and low-quality regions using the trimmomatic software (ver 0.38) (Bolger et al., 2014) with the following settings: LEADING:3, TRAILING:3, SLIDINGWINDOW:4:15, MINLEN:100.

The remaining high-quality sequences were aligned to an in-house genome assembly of parent DJ123 for the BC1F3 and to the publicly available genome assembly of a tropical japonica cultivar Azucena (NCBI BioProject PRJNA424001) for the BC1F5, using bwa software (ver 0.7.17) (Li and Durbin, 2009). Aligned reads were ordered, indexed and converted to BAM format with samtools (ver 1.9) (Li et al., 2009). Variants were extracted using bcftools software (ver 1.9) (Li et al., 2009). The extracted single nucleotide polymorphisms (SNPs) markers were filtered by vcftools software (ver 0.1.16) (Danecek et al., 2011) using the following settings: –min-meanDP 10 –max-meanDP 50 –max-missing 0.95 –minQ 20 –min-alleles 2 –max-alleles 2 for the BC1F3 population. For BC1F5 population, –max-meanDP 100 option was used due to deeper sequencing depth. Monomorphic and indel markers were further removed. For the BC1F3 population, missing alleles were imputed by BEAGLE software (ver 5.1) (Pook et al., 2020). Further removal of monomorphic and indel markers resulted in a total of 1578 markers in the BC1F3 population, which was further manually reduced to 222 based on minor allele frequency (> 0.1), physical positions and likelihood of genotyping error calculated by the R/qtl software (http://www.rqtl.org; Broman et al., 2003). For the BC1F5 population, SNPs within 20 bp of detected indel sites were removed. To minimize spurious heterozygous calls, heterozygous allele was defined only when >5 reads from each parent supported the genotype. Subsequently, missing alleles were imputed by the *k*-nearest neighbor imputation method using TASSEL 5 software (Bradbury et al., 2007; Money et al., 2015). Further removal of monomorphic and indel markers resulted in a total of 7398 genome-wide SNP markers in the BC1F5. These markers were reduced to 299 markers based on minor allele frequency (> 0.1) and physical distance.

### Genetic linkage map construction and QTL analysis

Above process retained 222 SNP markers for the BC1F3 and 299 SNP markers for the BC1F5. SNP calls were converted to the ABH-format, where “A’, ‘B’ and ‘H’ denote the donor DJ123 allele, recipient Nerica4 allele and heterozygote state, respectively. The genetic linkage map was constructed using R software package Rqtl and LinkageMapView (Broman et al., 2003; Ouellette et al., 2018). Markers were initially ordered based on their physical map positions but the final order was determined after applying the ripple function in Rqtl. Genetic distances were estimated in cM using the Kosambi option.

The software PLABQTL (http://www.uni-hohenheim.de/ipspwww/soft.html; Utz and Melchinger, 1996) was used for QTL mapping. Mean phenotypic values of 4 replicates with 3 sampled plants per replicate were used as the input. Putative QTL were detected by Composite Interval Mapping, in which the most influential markers based on an F-to-enter of 8.0 were used as cofactors in the analysis. For each trait, significance threshold was determined by 1000 times of permutation test at a significance level of 5% and 10%.

For the confirmation of detected QTL in the BC1F5, only lines homozygous at both flanking markers were selected. The effect of substituting Nerica4 (BB) alleles by DJ123 (AA) alleles was estimated based on mean phenotypic values of respective classes using Welch’s *t* test.

## Results

### Phenotypic variation

**Figure 1:**
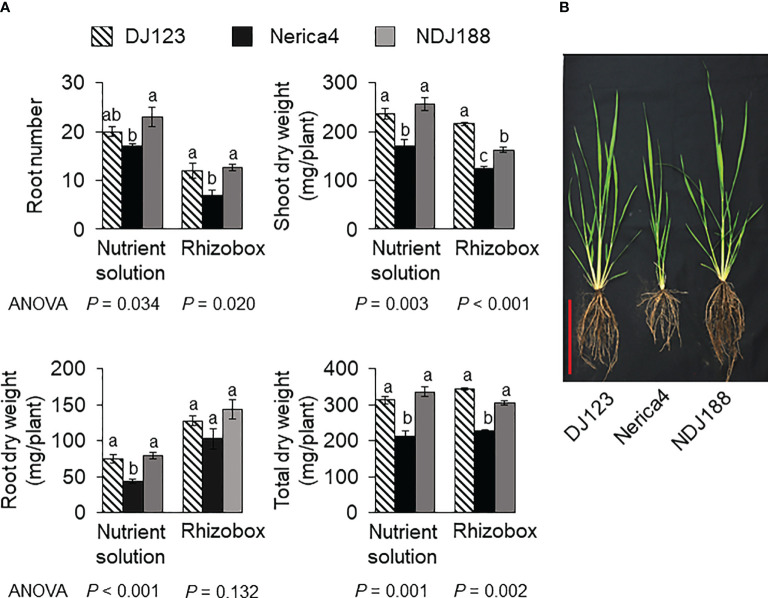
Phenotypic variation in parental genotypes DJ123, Nerica4, and NDJ188 grown under P deficient conditions. **(A)** Root number (RN), shoot dry weight (SW), root dry weight (RW) and total dry weight (TW) of plants grown for 35 d in low-P nutrient solution or in rhizoboxes filled with low-P field soil. Means and standard deviations are shown. One-way ANOVA was conducted for each experiment, and the resultant *p* values are shown. Different letters indicate significant differences (*p* < 0.05, Tukey’s HSD test). N=4 and 3 for nutrient solution and rhizobox experiment, respectively. **(B)** Representative plants grown for 60 d in a P deficient field at JIRCAS, Japan. Red scale bar indicates 20 cm.

**Figure 2:**
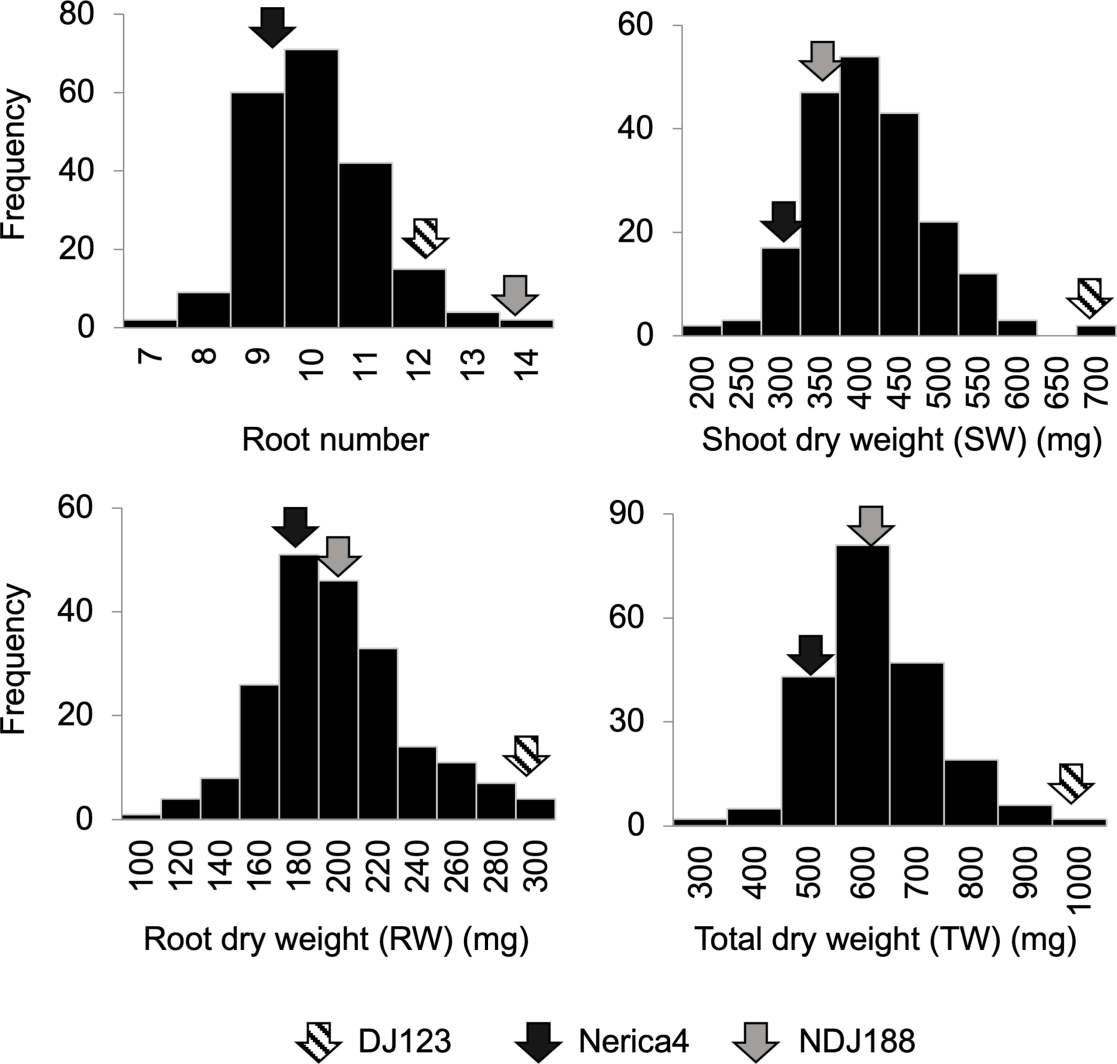
Frequency distribution of 201 BC1F3 lines for root number, shoot weight, root weight and total weight from a field experiment on low-P soil conducted at JIRCAS, Tsukuba, Japan in 2020. Arrows indicate the phenotypic values of parents (DJ123, Nerica4 and NDJ188).

The comparison of parents used to develop the QTL mapping population in nutrient solution, rhizobox and field experiments under P deficient conditions showed that P efficient donor DJ123 had significantly higher biomass compared to recurrent parent Nerica4 and that parental line NDJ188 resembled DJ123 in most traits (**Figure 1A**). Under field conditions the higher biomass was accompanied by a larger root system (**Figure 1B**). In the field experiment conducted in Japan and providing the phenotypic data for QTL mapping or the 201 BC1F3 lines, large variation was observed for the traits measured at 40 DAS (**Figure 2**). Root number varied by two-fold from 7 to 14 crown roots and root biomass varied from 100 mg to 300 mg per plant with 60 lines being superior to the two parents Nerica4 and NDJ188. Similar 3-fold variation was observed for shoot weight and total biomass weight. The distribution of the four traits (RN, RW, SW and TW) was close to a normal distribution. Nerica4 had below average values for all traits whereas DJ123 was among the best performers for biomass traits and NDJ188 for root number (**Figure 2**). Correlations between biomass traits were high; Pearson’s correlation coefficient ranged from *r* = 0.75 (*P* < 1.0 × 10-15) between root and shoot biomass to *r* = 0.96 (*P* < 1.0 × 10-15) for shoot and total biomass (**Supplementary Table S1**). Root number (RN) had moderate positive correlations of around *r* = 0.5 (*P* = 8.1 × 10-13) with biomass traits.

### QTL detection

A linkage map containing 222 SNP markers was generated for the BC1F3 mapping population, by aligning RAD-seq reads to the parental DJ123 reference sequence and subsequent reduction of very closely linked markers (**Supplementary Figure S1**). Since this is a backcross population of Nerica4-derived NDJ188 to Nerica4, the linkage map is characterized by large non-recombinant blocks. This resulted in very small recombinant regions on chromosomes 3, 4, 7, 11, and 12.

**Figure 3:**
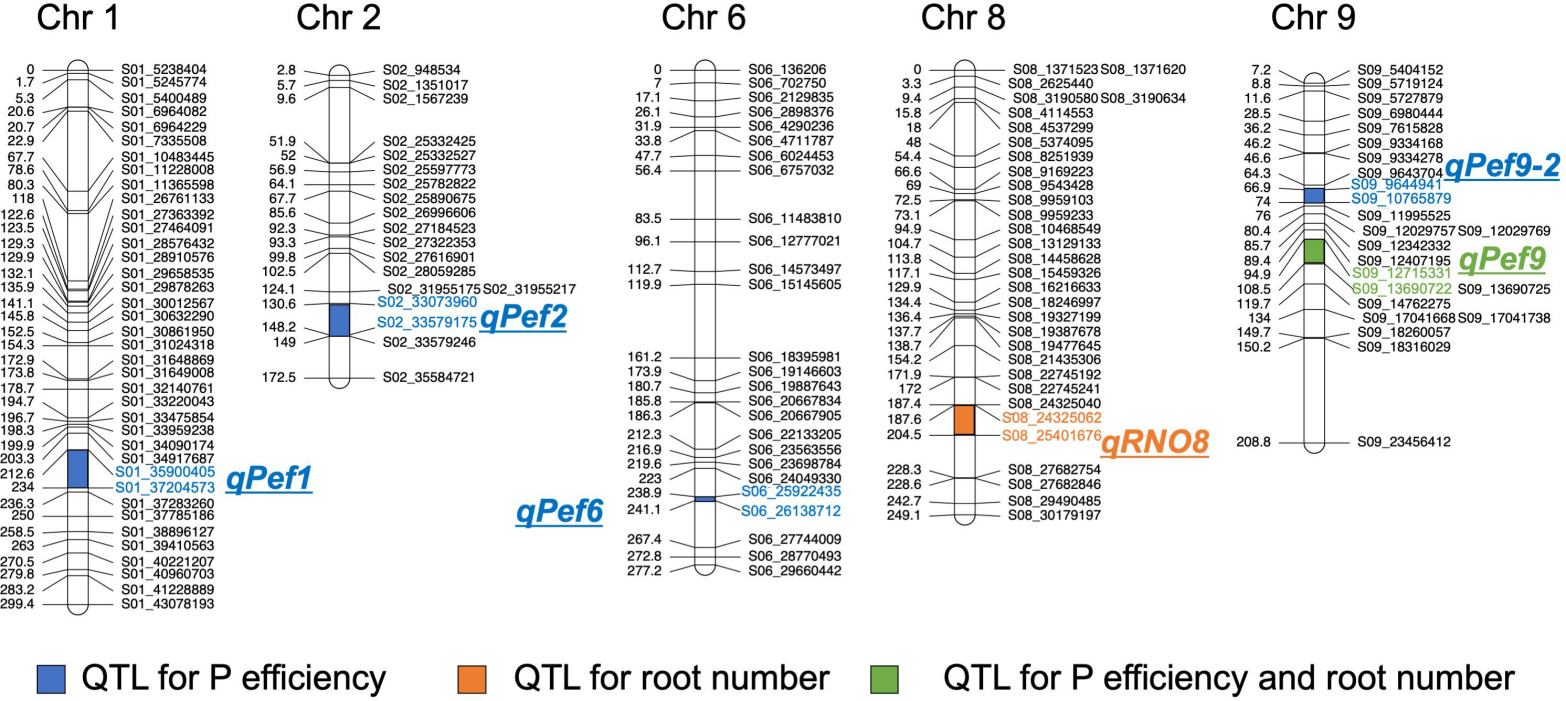
Chromosomal positions of the QTL detected in the BC1F3 population. Genetic distance and name for each marker are shown for chromosomes 1, 2, 6, 8, and 9.

A total of 10 putative QTL were detected for the traits RN, SW, RW and TW and several traits mapped to the same locus, indicating that only 6 distinct putative QTL were identified. One QTL affecting the P efficiency traits RN, RW and TW was detected on chromosome 9 (*qPef9* – for P efficiency) within the same interval (**Figure 3**, **Table 1**), whereas two QTL overlapped for SW and TW on chromosomes 1 (*qPef1*) and 6 (*qPef6*). Two QTL were detected for RN; qRNO8 and *qPef9*, jointly explaining 23.4% of the variation for RN among the 201 backcross lines. Alleles increasing RN were from donor DJ123. In addition to *qPef9*, one minor locus was detected for RW on chromosome 2 (*qPef2*) and this locus was the only one for which Nerica4 contributed the positive allele (**Table 1**). Variation in SW was attributed to three putative QTL on chromosomes 1 (*qPef1*), 6 (*qPef6*) and 9 (*qPef9-2*), together explaining 46.8% of the variation for this trait. Of the three putative QTL detected for TW, two coincided with QTL for SW (*qPef1*) and (*qPef6*) and one with a QTL for RW and RN (*qPef9*) (**Figure 3**).

**Table 1:** Putative QTL for root number and biomass traits based on phenotypic data generated for the BC1F3 population from a field experiment conducted on highly P deficient soil in Japan.

Trait	QTL name	Chromosome	Nearest marker^a^	Position^b^ (cM)	Support interval^c^	LOD^d^	R^2^(%)^e^	Additive effect^f^	Positive allele
Root Number	*qRNO8*	8	C8M27	200	186-216	5.55**	11.9	-0.36	DJ
Root Number	*qPef9*	9	C9M16	96	86-110	5.35**	11.5	-0.37	DJ
Total							23.4		
Root Weight	*qPef2*	2	C2M16	136	126-148	4.46*	9.7	1.24	N4
Root Weight	*qPef9*	9	C9M16	96	88-108	5.91**	12.7	-1.23	DJ
Total							22.4		
Shoot weight	*qPef1*	1	C1M29	216	208-232	7.11**	15	-3.37	DJ
Shoot weight	*qPef6*	6	C6M23	242	238-254	7.64**	16.1	-3.6	DJ
Shoot weight	*qPef9-2*	9	C9M9	66	62-70	7.48**	15.7	-2.65	DJ
Total							46.8		
Total Weight	*qPef1*	1	C1M29	218	208-232	6.48**	13.8	-6.8	DJ
Total Weight	*qPef6*	6	C6M23	242	238-264	6.33**	13.5	-4.07	DJ
Total Weight	*qPef9*	9	C9M16	94	88-102	9.3**	19.2	-4.66	DJ
Total							46.5		

a Nearest marker from the peak of the QTL. Marker name indicates the chromosome and the relative position of the markers in the chromosome.

b Genetic position of the putative QTL in cM.

c Confidence interval of the location of the putative QTL in cM.

d Logarithm of the odds score of QTL detected using the threshold of LOD, based on the 1000 times of permutation test. * and ** indicate significance level at 10% (LOD> 4.22 for RW) and 5% (LOD> 4.83, 4.72, 4.72 and 4.78 for RN, RW, SW and TW), respectively.

e Percentage of the variation explained by the QTL.

f Additive effect: negative value indicates the genotype from the parent DJ123 toward increasing the trait value.

### QTL interactions and effects in BC1F3

To what extent interactions between two detected QTL affected phenotypic values in the BC1F3 was explored in **Supplementary Tables S2** – **S4** where AA and BB stand for the DJ123 and Nerica4 allele, respectively. The interaction for root number QTL *qPef9* with *qRNO8* indicated the presence of small non-additive effects, since having DJ alleles at both loci increased root number more than expected from the single locus effects (**Supplementary Table S2**). On the other hand, the interaction between root biomass QTL (*qPef9* and *qPef2*) was not significant (**Supplementary Table S3**). Among shoot biomass QTL (*qPef9-2*, *qPef1*, and *qPef6*) the interaction between *qPef9-2* and *qPef1* was not significant but the other 2 combinations yielded significance (**Supplementary Table S4**). The interaction between the total biomass QTL (*qPef6* and *qPef9*) was significant, with AAAA types having a larger advantage (+23 to +26%) over double-negative BBBB, than expected from a single allele change (+5 to +8% in AABB or BBAA).

### Confirmation of QTL effects in Madagascar and Japan using derived BC1F5 lines

To genotypically characterize the BC1F5 lines derived from the original BC1F3 lines, a new set of 299 SNP markers was utilized from a new RAD-seq analysis. Their average segregation ratio was 44.3% DJ123 allele (AA), 39.5% Nerica4 allele (BB) and 16.1% heterozygotes. Six distinct QTL had been identified in the initial BC1F3 experiment and to what extent their effects can be confirmed was examined in field experiments with BC1F5 lines selected for homozygosity using markers flanking the QTL position.

Locus *qPef9* had strong positive effects on TW, SW and RN in the initial experiment in Japan, and in both confirmatory experiments, the DJ123 allele improved performance, although effects were smaller than anticipated (**Table 2**), except for RN where the effect of the AA allele is significantly higher than the BB allele. Interestingly the largest effect was observed for SW, for which a different QTL (*qPef9-2*) on chromosome 9 had been mapped in the BC1F3 (**Figure 3**). This second QTL on chromosome 9 generally had very strong effects in Madagascar, increasing SW and TW by 23.8% and 18.2%, respectively, but also significantly increasing RW and RN (**Table 2**). Remarkably, the two QTL observed on chromosome 9 do have similar and significant effects in all the traits in Madagascar site and only on RN in Japan site. Two other QTL could be confirmed in Japan but not in Madagascar, namely *qPef6* that increased RN, SW and TW and qRNO8 that increased RN. The remaining QTL, *qPef1* and *qPef2*, had only minor influence in the BC1F5 lines in either site, and did not yield significant difference (**Table 2**),

**Table 2:** Effect of the positive alleles at detected QTL on average phenotypic values of selected homozygote lines in the BC1F5 generation.

		Madagascar					Japan				
QTL	Allele	RN	RW	SW	TW	(n)^a^	RN	RW	SW	TW	(n)^a^
*qPef1*	AA	20.3	172	**654**	**809**	90	13.4	213	**220**	**433**	87
	BB	21	186	**629**	**834**	19	13.9	208	**213**	**420**	20
	%	-3.4	-8.1	3.8	-3.1		-3.7	2.3	3.2	3.0	
*qPef2*	AA	20.5	**170**	631	796	57	14	**211**	218	429	52
	BB	20.2	**171**	660	800	58	13.1	**211**	219	430	57
	%	1.5	-0.6	-4.6	-0.5		6.4	0.0	-0.5	-0.2	
*qPef6*	AA	21	180	**678**	**819**	59	13.8	218	**226**	**444**	52
	BB	20.1	162	**640**	**807**	42	13	209	**200**	**409**	45
	%	4.3	10.0	5.6	1.5	28.8	5.8*	4.1	11.5*	7.9*	
*qRNO8*	AA	**20.2**	174	691	811	47	**13.9**	215	222	437	41
	BB	**20.9**	181	653	842	60	**13.3**	213	223	435	57
	%	-3.5	-4.0	5.5	-3.8		4.3*	0.9	-0.5	0.5	
*qPef9-2*	AA	21.3	185	**711**	878	74	14	217	**225**	441	74
	BB	19.5	160	**542**	718	42	12.9	203	**212**	415	38
	%	8.5*	13.5*	23.8*	18.2*		7.9*	6.5	5.8	5.9	
*qPef9*	AA	**21.3**	**187**	708	**885**	68	**14.1**	**217**	224	**442**	69
	BB	**19.4**	**160**	572	**731**	41	**12.8**	**208**	213	**420**	38
	%	8.9*	14.4*	19.2*	17.4*		9.2*	4.1	4.9	5.0	

a Number of the homozygote lines for each allele, based on both flanking markers.

*Significant difference between AA and BB allele, with a significance level, 5% (Welch’s *t* test).

Percent values indicate the difference between AA (DJ123) and BB (Nerica4) genotypic classes, using flanking markers of **Figure 3** to define QTL positions (corresponding markers in the BC1F5 are shown in **Supplementary Table S5**). Traits highlighted in bold print are those for which a particular QTL had been identified in the BC1F3.

**Figure 4:**
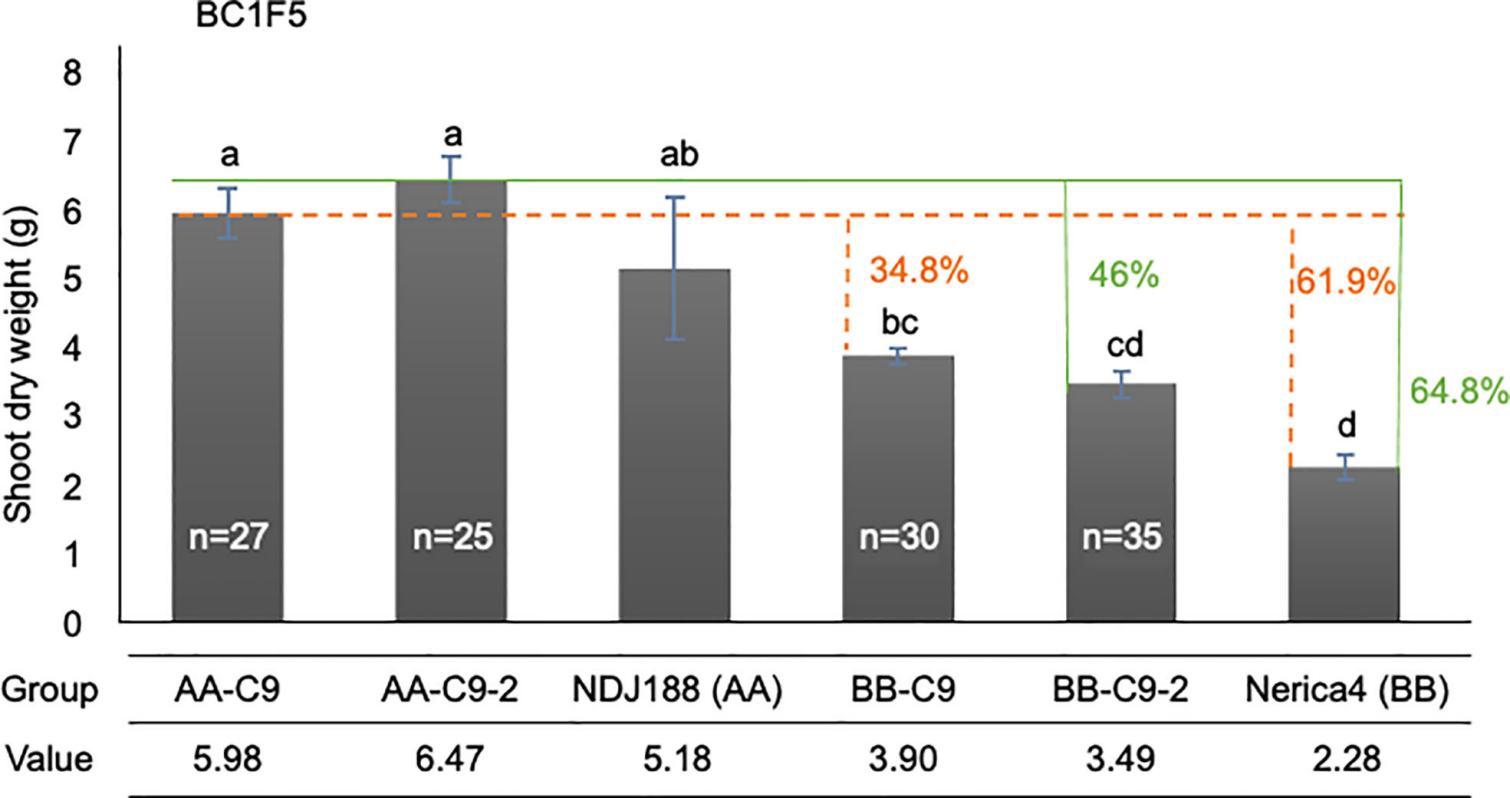
Confirmation of the QTL *qPef9-2* and *qPef9* on shoot weight trait in Madagascar at 70 DAS. Solid green lines point to the effect having AA alleles at *qPef9-2* compared to either the BB alleles in BC1F5 lines or to recurrent parent Nerica4. Dotted orange lines point to the effects of having AA alleles at *qPef9*, compared to BB alleles or parent Nerica4. Different letters indicate significant differences (*p* < 0.05, Tukey’s HSD test). “n” indicates the number of lines classified into each genotype group. For each genotype, including parent lines, the data are mean value of 3 replications, each of which was obtained from 5 individual plants.

The initial QTL mapping study identified two apparently distinct QTL on chromosome 9 controlling different traits. However, confirmatory trials in Madagascar placed this distinction in question as *qPef9* and *qPef9-2* affected traits similarly, but with stronger effects for *qPef9-2* compared to *qPef9* on SW and TW in Madagascar. The general similarity of effects of both chromosome 9 QTL with stronger effects in *qPef9-2* were confirmed by some additional plant sampling for SW during the late vegetative stage in Madagascar. Results are summarized in **Figure 4**, showing highest SW being achieved by lines homozygous for DJ123 at *qPef9-2*, which outperformed their Nerica4 allele counterparts by 46% and parent Nerica4 by 64.8%. Lines homozygous for DJ123 at *qPef9* also significantly outperformed their Nerica4 allele counterparts (+34.8%) and parent Nerica4 (+61.9%).

## Discussion

Upland rice has the potential to play an important role in satisfying the increasing demand for rice in SSA, but two recent global trends make improvements in upland rice productivity even more precarious. These are the increasing costs of fertilizers that place P fertilizer purchases out of reach of small-holder farmers (Tsujimoto et al., 2019), and global climate change leading to less predictable rainfall patterns that more strongly affect rice as a relatively drought-sensitive crop (Shaw et al., 2011). These combined effects are likely to particularly affect upland rice crop establishment on the highly weathered tropical soils such as Oxisols that represent the typical soils in upland rice farming regions of Madagascar (Rabeharisoa, 2004). Such soils are characterized by strong sorption and low mobility of P and with less frequent rainfall, P mobility and therefore plant availability will decrease further because P mobility decreases sharply with reduced soil moisture (Marin et al., 2021).

Rapid seedling root development is a key trait to assure P uptake quickly reaches sufficiently high rates to support crop establishment after seed-P reserves have been depleted (Pariasca-Tanaka et al., 2015) and the donor (DJ123) used in developing the QTL mapping population was found to be far superior to the recurrent parent Nerica4, which is characterized by rather slow seedling growth and root development when P is growth-limiting (Wissuwa et al., 2020). Identifying QTL for rapid seedling root development and seedling biomass accumulated under P deficiency in the Nerica4 genetic background could therefore be a potentially important step towards improving these traits in the otherwise well-adapted variety Nerica4.

Root trait differences between both parents have been explored in detail but apart from slightly more pronounced lateral root branching in DJ123, root system architecture was remarkably similar between both parents, which included a rather similar distribution at soil depth (Mori et al., 2016; Wissuwa et al., 2020). Despite concentrating most roots in the plow layer, neither parent was classified as having a shallow root system when compared to other rice genotypes more adapted to flooded conditions (Mori et al., 2016) and the detailed evaluation of the DJ123 confirmed an intermediate root type with a root angle distribution peaking around 30° from the soil surface (Gonzalez et al., 2021). Considering the similarity of root systems between parents, but the very pronounced difference in early seedling vigor, this study focused on this latter trait.

### QTL mapping and confirmation of QTL effects

QTL mapping was conducted in Japan on the same soil used to characterize the parental accessions and Nerica4 was confirmed to lack early seedling vigor while the majority of BC1F3 lines surpassed the recurrent parent. We detected 6 distinct QTL associated with traits RN, RW, SW, and TW. Three QTL detected for SW and TW on chromosomes 1, 6 and 9 jointly explained 47% of the variation for the respective traits. Given the tight correlation (*r* = 0.96) between SW and TW, it was expected that QTL for both biomass traits would overlap, as in the case of *qPef1* and *qPef6*, and possibly for the neighboring chromosome 9 loci *qPef9* and *qPef9-2* that may represent one large peak rather than two distinct loci, as to be discussed in a following paragraph.

The confirmation with a smaller set of lines in the subsequent BC1F5 generation was conducted in Japan and in the target upland rice region of central Western Madagascar where Nerica4 is a commonly grown variety. While most small-effect QTL could not be confirmed, *qPef9-2* and to a lesser extent *qPef9* showed very positive effects in Madagascar, from a 8.5% increase in crown root number to a 23.8% improvement of SW in 40-day old plants. The positive effect increased further over time to 46% higher SW at the late vegetative stage, indicating that QTL effects are not limited to the seedling stage. Instead, good seedling development under P deficiency was expected to lay the foundation for subsequent crop development and this appears to have been the case here. To what extent better root development was the driving factor for plant growth on these low-P soils cannot be answered with certainty, however, that chromosome 9 significantly increased RN in all 3 experiments may indicate that root development is a key process affected by *qPef9/qPef9-2*.

A question to be resolved through subsequent fine-mapping is whether chromosome 9 harbors two distinct and closely linked loci or whether *qPef9* and *qPef9-2* should in fact be considered as a single locus spanning the region from 9.19 to 12.92 Mb on chromosome 9. However, based on the evidence obtained in the current study, we may cautiously conclude that a single locus is the most likely scenario based on the confirmatory data obtained in Madagascar that no longer distinguishes shoot from root-related QTL effects. Furthermore, using the seq/s command in PlabQTL to return LOD estimates in 10 cM intervals for the 56 – 106 cM region on chromosome 9 indicated that the entire 66 – 106 cM region was above LOD 3.0 for biomass traits, with peaks at 66 and 96 cM (**Supplementary Figure S2**) but without a clear drop in significance between the peaks. Last but not least, a QTL region of 3.72 Mb size is not excessively large. Evaluation of a large number of F2 plants derived from the cross between the recurrent parent Nerica4 and a line containing this entire region would allow us to resolve whether a single or two distinct QTL exist through fine-mapping, which would also be the first step towards identifying the underlying causal gene(s).

### Supporting evidence from other QTL mapping studies

Several QTL associated with tolerance to P deficiency have been detected in rice (Wissuwa et al., 1998; Shimizu et al., 2004; Koide et al., 2013; Kale et al., 2021), of which the most influential appears to be the *Pup1* locus on chromosome 12 (Wissuwa et al., 1998; Wissuwa et al., 2002). Among these studies, Shimizu et al. (2004) detected a QTL for P deficiency-induced root elongation under a hydroponic condition on chromosome 6 that appears to be overlapping with *qPef6* detected here for SW and TW. The same study also detected QTL for shoot dry weight under P deficiency and P deficiency-induced changes in shoot P content at very close positions, suggesting that root growth under P deficiency is linked with P uptake and shoot growth under P deficient conditions. Further investigations are necessary to determine if *qPef6* is related with root elongation and if such trait is relevant with P uptake and shoot growth under P-deficient field conditions. Another QTL detected by Kale et al. (2021) was located on chromosome 8, but seemingly towards the opposite end of chromosome from qRNO8 detected here.

For the main QTL *qPef-9/qPef9-2* affecting root number and biomass traits, two studies detected QTL at similar positions in relation to biomass production in pot experiments under fertilized well-watered upland conditions. Mu et al. (2003) identified *rdw9* between 12.41 – 18.49 Mb on chromosome 9 in the Yuefu x IRAT 109 population, explaining 18.5% of the variation for root dry weight. A second QTL at the very similar interval of 9.78 – 12.41 Mb was detected in the IAC165 x Co39 population, explaining 5.5% of the variation for seedling shoot weight of plants (Courtois et al., 2003). Thus, a region containing *qPef-9* and *qPef9-2* might be responsible for root growth under upland conditions irrespective of P conditions, which improves biomass production under P deficient conditions by enhancing P uptake as a consequence of extending the effective soil volume for P uptake.

Resolving to what extent this presumed chain of events is correct would be highly desirable as this would allow fine-tuning strategies for future fine-mapping and candidate gene identification. For a QTL affecting root growth screening would be possible in nutrient solution or greenhouse experiments, providing data less prone to environmental effects. In contrast, a QTL related to P uptake processes at the soil-root interphase would require screening in soil, and the more positive effect in Madagascar would suggest that fine-mapping should ideally be done under these local conditions. A third possibility is that *qPef9* may be due to processes related to internal P efficiency or P allocation between shoot and root, and such effects are notoriously difficult to phenotype in field experiments, thus would ideally require very specific screening experiment during fine-mapping. In order to provide insights into these alternative underlying mechanisms physiological studies should be conducted using contrasting lines identified in experiment 3.

## Conclusions

We have detected and confirmed a strong QTL on chromosome 9 that increased crown root number and improved plant growth under P deficient conditions in Madagascar. Lines with the donor DJ123 alleles at this locus had more than 60% higher shoot biomass compared to recurrent parent Nerica4. The QTL identified here are practically relevant because they were detected and confirmed in the target genetic background of the local variety Nerica4. Superior performing lines of the mapping population can therefore enter multi-local yield trials in the target region.

## Data availability statement

The data presented in the study are deposited in the NCBI SRA repository, accession number PRJNA870681 (https://www.ncbi.nlm.nih.gov/sra/PRJNA870681) and PRJNA870975 (https://www.ncbi.nlm.nih.gov/sra/PRJNA870975).

## Author contributions

HR, DL, and JPT carried out field and greenhouse experiments and collected data. JPT and HT prepared DNA and sequencing library. YU conducted bioinformatic analyses and prepared markers. HR, DL, and MW conducted data analysis. HR and DL wrote the manuscript draft and prepared figures. MW conceived the research and wrote the manuscript. LR, TR, and MW supervised experiments. All the authors read and approved the manuscript.

## Funding

This research was supported by the Science and Technology Research Partnership for Sustainable Development (SATREPS), Japan Science and Technology Agency (JST)/Japan International Cooperation Agency (JICA)—Grant No. JPMJSA1608.

## Acknowledgments

The authors thank field technical staff members of JIRCAS and at local farmers’ site in Madagascar for management of the field and assistance in plant phenotyping. The authors thank M. Yonemoto for preparation of DNA samples. We acknowledge Dr. Nani Drame (Former Africa Rice Center staff) for sharing seeds of the Nerica4 x DJ123 mapping population with FOFIFA.

## Conflict of interest

The authors declare that the research was conducted in the absence of any commercial or financial relationships that could be construed as a potential conflict of interest.

## Publisher’s note

All claims expressed in this article are solely those of the authors and do not necessarily represent those of their affiliated organizations, or those of the publisher, the editors and the reviewers. Any product that may be evaluated in this article, or claim that may be made by its manufacturer, is not guaranteed or endorsed by the publisher.
