# Corrigendum: QTL mapping for early root and shoot vigor of upland rice (*Oryza sativa* L.) under P deficient field conditions in Japan and Madagascar

**DOI:** 10.3389/fpls.2022.1123825

**Published:** 2023-01-17

**Authors:** Harisoa Nicole Ranaivo, Dinh Thi Lam, Yoshiaki Ueda, Juan Pariasca Tanaka, Hideki Takanashi, Landiarimisa Ramanankaja, Tantely Razafimbelo, Matthias Wissuwa

**Affiliations:** ^1^ Rice Research Department, National Center for Applied Research on Rural Development (FOFIFA), Antananarivo, Madagascar; ^2^ Crop, Livestock and Environment Division, Japan International Research Center for Agricultural Sciences (JIRCAS), Tsukuba, Japan; ^3^ Institute of Agricultural Science for Southern Vietnam (IAS), Ho Chi Minh City, Vietnam; ^4^ Graduate School of Agricultural and Life Sciences, The University of Tokyo, Tokyo, Japan; ^5^ Laboratory of Radioisotopes, University of Antananarivo, Antananarivo, Madagascar

**Keywords:** phosphorus deficiency, crop establishment, crown root number, NERICA, seedling vigor

In the published article, there was a mistake in the **Materials and Methods** section. We omitted the source (Africa Rice Center) of the original material from which the QTL mapping population used in this study was developed and misstated the purpose for developing that original material. It was not targeting Madagascar specifically but all P deficient environments in Sub-Saharan Africa.

A correction has been made to **Materials and Methods**, *Plant Material*, 1^st^ paragraph. This sentence previously stated:

“An initial breeding population targeting P deficient upland environments in Madagascar had been developed from a cross of P efficient genebank accession DJ123 with P inefficient upland variety Nerica4.”

The corrected sentence appears below:

“An initial breeding population targeting P deficient upland environments in SSA had been developed from a cross of P efficient genebank accession DJ123 with P inefficient upland variety Nerica4 by the Africa Rice Center and shared with FOFIFA for further advancement and evaluation in Madagascar.”

A correction has also been made to Acknowledgments. The sentence below has now been added:

“We acknowledge Dr. Nani Drame (Former Africa Rice Center staff) for sharing seeds of the Nerica4 x DJ123 mapping population with FOFIFA.”

The authors apologize for these errors and state that this does not change the scientific conclusions of the article in any way. The original article has been updated.

