# Supplementary material for: QTL mapping for early root and shoot vigor of upland rice (Oryza sativa L.) under P deficient field conditions in Japan and Madagascar
Source: Front Plant Sci. 2022 Oct 24;13:1017419. doi: 10.3389/fpls.2022.1017419 (PMC9637880; doi:10.3389/fpls.2022.1017419)
Supplement: Supplementary file 2 [file DataSheet_2.docx]

**Supplementary Figure S1:** Linkage map based on 222 SNP markers in the BC1F3 population derived from a backcross of NDJ188 to recurrent parent Nerica4. Genetic distance and name for each marker are shown.

**Supplementary Figure S2:** LOD peak curves for QTL detected on chromosome 9 for traits RN, RW, SW, TW based on the analysis conducted in PLABQTL.
